# Enhancement of mitochondrial biogenesis with polyphenols: combined effects of resveratrol and equol in human endothelial cells

**DOI:** 10.1186/1742-4933-10-28

**Published:** 2013-07-11

**Authors:** Sergio Davinelli, Nadia Sapere, Manuela Visentin, Davide Zella, Giovanni Scapagnini

**Affiliations:** 1 Department of Medicine and Health Sciences, University of Molise, Campobasso 86100, Italy; 2 Paladin Pharma S.p.A., R&D Division, Torino 10126, Italy; 3 Institute of Human Virology, Department of Biochemistry and Molecular Biology, University of Maryland-School of Medicine, Baltimore, MD 21201, USA

## Abstract

Emerging evidence suggests that combinatorial action of numerous biologically active compounds may be a valuable source in a variety of therapeutic applications. Several nutraceuticals have demonstrated to augment the efficacy of pharmacological approaches or provide physiological benefit to improve age-related decline. Recently, the possibilities of anti-ageing interventions have multiplied also to ameliorate the mitochondrial alterations in ageing-associated diseases. In this report, we approached a novel treatment strategy by combining two bioactive dietary constituents (resveratrol and equol) to determine their effect on mitochondrial function. Taking into account that the biological activities of resveratrol and equol has been observed in a wide range of biological processes, they were selected to examine whether combining them would be more effective to modulate mitochondrial function. In HUVEC cells our results demonstrate that the co-administration of these natural products increased mitochondrial mass and mitochondrial DNA content. Additionally, combined use of both compounds increased SIRT1 enzymatic activity and induced mitochondrial biogenesis factors such as PGC1-α, TFAM and NRF-1. Therefore, identification of this novel synergism may provide a new perspective for future treatments aiming to modulate the mitochondrial activity with implications in maintaining endothelial function which is crucial in the regulation of immune response. Further studies to discover the molecular details of this crosstalk and to identify new combinations of active compounds affecting the mitochondrial function will be extremely beneficial to prevent mitochondrial decline.

## Background

Ageing process is characterized by a general decline in cellular activity and it is also associated with a decrease in mitochondrial function correlated to the onset and progression of age-related pathologies [1]. Mitochondria play an essential role in ageing and human umbilical vein endothelial cells (HUVEC) have been established as a cellular model to follow mitochondrial dysfunction during the ageing process [2,3]. Consistent with this notion, it is imperative to uncover the molecular mechanisms underlying the regulation of the decline of mitochondria in order to identify potential therapeutic targets. Several regulatory factors are implicated in the modulation of mitochondrial function [4] and peroxisome proliferator-activated receptor (PPAR) γ – coactivator-1 (PGC1-α) has emerged as a master regulator for the mitochondrial transcription and translation machinery [5]. Moreover, PGC1-α appears to act as a central coordinator of multiple transcription factors [6]. In particular, it has been shown that PGC1-α is able to strongly interact and co-activate the nuclear respiratory factor 1 (NRF-1) [7]. Furthermore, NRF-1 is implicated in the interaction with several mitochondrial genes including the mitochondrial transcription factor A (TFAM), one of the most important mammalian transcription factors for mitochondrial DNA (mtDNA) [8]. Noteworthy, recent evidence has highlighted NAD^+^-dependent protein deacetylase sirtuin 1 (SIRT1) as a critical factor for the regulation of mitochondrial function. Indeed, SIRT1 with PGC1-α and its regulatory circuit have been recognized to have a direct involvement in the control of mitochondrial biogenesis and metabolism [9]. Therefore, PGC1-α constitutes an energy sensing cellular platform that controls mitochondrial function and its network provides a link between mitochondria and ageing that may be useful as an anti-ageing strategy. Recently, synergy assessment has become a key area in medicine research in order to enhance efficiency of treatments and affect not only one single target, but several targets. Numerous nutraceuticals have been found to target and attenuate the progression of age-related dysfunction [10,11]. Currently, there are a variety of dietary strategies to ameliorate mitochondrial function in ageing [12]. For instance, among isoflavones (members of the class of flavonoids) equol (the main active product of daidzein metabolism) has recently attracted scientific interest because of its powerful antioxidant activity with implications in treating age-related diseases [13], however its influence on mitochondria is still poorly understood. On the contrary, resveratrol is a naturally occurring polyphenol with wide-ranging health benefits including well-established properties in promoting mitochondrial biogenesis during ageing [14]. The present study aims to investigate the effects of resveratrol and equol on mitochondrial biogenesis using the two compounds individually and in combination.

## Materials and methods

### Cell cultures

HUVEC were obtained from Lonza and maintained in endothelial basal medium (EBM-2) supplemented with growth factors (Lonza, Walkersville, MD). Cells were grown at 37°C in 5% CO_2_ and serial passages were performed when the cells reached a 80% confluence. As described by Grillari *et al.*[15] at around 30 passages when the cells exhibited the irreversible growth arrest, they were used for the experimental procedures. HUVEC cells were treated with resveratrol (trans-3, 4, 5,-trihydroxystilbene) (purity 98%) purchased from Cayman Chemical Company (Ann Arbor, MI) and equol ((3S)-3-(4-Hydroxyphenyl)-7-chromanol) (purity 98%) purchased from INDOFINE Chemical Company (Hillsborough, NJ).

### MitoTracker® Red staining

The HUVEC cells were subdivided into three groups, which were treated, as previously tested [16], with resveratrol, 10 μM for 48 hours, equol 10 μM [17] for 48 hours, and with the composition containing resveratrol and equol 10 μM for 48 hours, respectively. The mitochondrial mass in HUVEC was determined selectively loading mitochondria with Mitotracker fluorescent red dye (Invitrogen, Carlsbad, CA). Fluorescent calcein (green) and Hoechst 33258 (blue) dyes were used to stain the cytoplasm and the nuclei, respectively. Optical sections of HUVEC were captured at ×60-magnification and the mitochondrial density-area was calculated with respect to cytoplasmatic volume using Zeiss AxioVision imaging software (Zeiss, Oberkochen, Germany). Only cells with intact cytoplasmatic calcein stain were included in the analysis.

### Quantitative real-time PCR for mitochondrial DNA

Total DNA was extracted from HUVEC cells using the DirectPCR lysis reagent (Viagen Biotech, Los Angeles, CA). The number of mtDNA copies was quantified by qRT-PCR according to the protocols of Adabbo *et al.*[18]. Two different housekeeping genes were used (cytochrome oxidase III and β-actin) for normalization. mtDNA per nuclear genome was calculated as the ratio of cytochrome oxidase III (mitochondrial) DNA to β-actin (nuclear) DNA. Quantification was performed using the ΔΔCT method.

### SIRT1 activity assay

Nuclear SIRT1 activity was evaluated in cells treated with resveratrol, equol and resveratrol + equol as described previously by Ferrara *et al.*[19]. We measured SIRT1 using a deacetylase fluorometric assay kit (Sir2 Assay Kit, CycLex, Ina, Nagano, Japan). The final reaction mixture (100 μL) contained 50 mM Tris–HCl (pH 8.8), 4 mM MgCl2, 0.5 mM DTT, 0.25 mA/mL Lysyl endopeptidase, 1 μM Trichostatin A, 200 μM NAD, and 5 μL of extract nuclear sample. All determinations were performed in triplicate.

### Measuring the mRNA expression of mitochondrial biogenesis factors via qRT-PCR

The qRT-PCR technique was used to determine the effect of resveratrol, equol and of the mixture of the two compounds (resveratrol + equol) (10 μM for 24 h) on mRNA expression of mitochondrial biogenesis factors such as Nrf-1, TFAM and PGC1-α in HUVEC cells using Light Cycler technology (Roche Molecular Biochemicals, Mannheim, Germany). The total RNA was isolated with Mini RNA isolation II Kit (Zymo Research, Orange, CA) and was reverse transcribed using SuperScript III RNase H-free reverse transcriptase (Invitrogen, Carlsbad, CA). Efficiency of the PCR reaction was determined performing dilution series of a standard sample. The housekeeping gene hypoxanthine phosphoribosyltransferase (HPRT) was used for internal normalization and quantification was performed using ΔΔCT method.

### Statistical analysis

Differences between various treatments were analysed by unpaired Student’s t-tests with P values <0.01 considered highly significant and P < 0.05 considered significant.

## Results and discusssion

### Combination of resveratrol and equol induces mitochondrial biogenesis in endothelial cells

**Figure 1:**
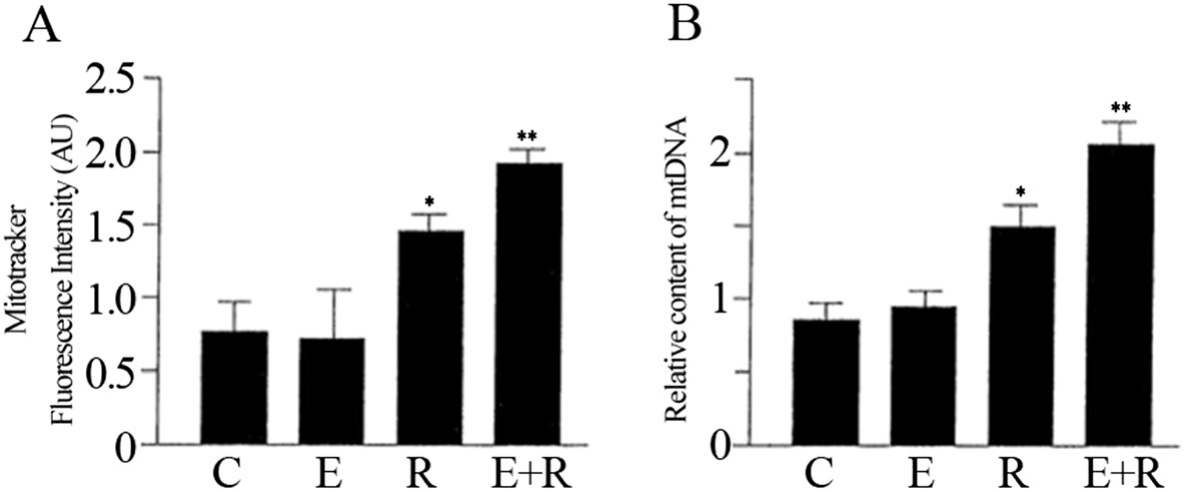
**The combined treatment of resveratrol and equol strongly increased the number of mitochondria in HUVEC cells. (A)** Mitotracker fluorescent intensities were analysed to assess the mitochondrial biogenesis. **(B)** Relative mitochondrial DNA (mtDNA) content was estimated by qRT-PCR. Representative data of at least 3 experiments each performed in triplicate. (*= P < 0.05, **= P < 0.01). C: control; E: equol; R: resveratrol; E+R: equol + resveratrol.

In the current study, we examined the combined treatment of resveratrol and equol on HUVEC cells to identify their effects on the mitochondrial biogenesis. In particular, the primary goal of this investigation was to determine whether combining resveratrol and equol would increase the expression of key factors involved in mitochondrial biogenesis. Several studies were conducted showing the synergistic effect of resveratrol with different compounds [20] but the combination with equol has never been tested on the mitochondrial function. The Mitotracker staining showed that mitochondria were located in the perinuclear region in HUVEC (data not shown). Treatment with resveratrol increased significantly the density-area ratio in Mitotracker-labeled endothelial cells as compared to the cytoplasmatic volume (Figure 1A).

Indeed, the analysis of Mitotracker intensity showed that resveratrol induced an increase in mitochondrial mass compared to non-treated cells (Figure 1A). Equol alone was not effective in terms of augmenting the mitochondrial mass, however the combined treatment (resveratrol + equol) was more effective respect to resveratrol alone (Figure 1A). It is important to point out that mitochondrial dysfunction tend to induce a wide range of adaptations of nuclear gene expression, named the retrograde response [21]. Typical of this adaptive process (mitohormesis) [22-24] is the up-regulation of mitochondrial biogenesis [25]. A robust adaptive response may further explain the increase in mitochondrial mass.

The enhanced mitochondrial biogenesis in cells treated with both compounds simultaneously was also confirmed by the increased cellular mtDNA content (Figure 1B). Overall, we found that co-treatment with resveratrol and equol positively affect mitochondrial biogenesis.

### Induction of SIRT1 by the association of resveratrol and equol

**Figure 2:**
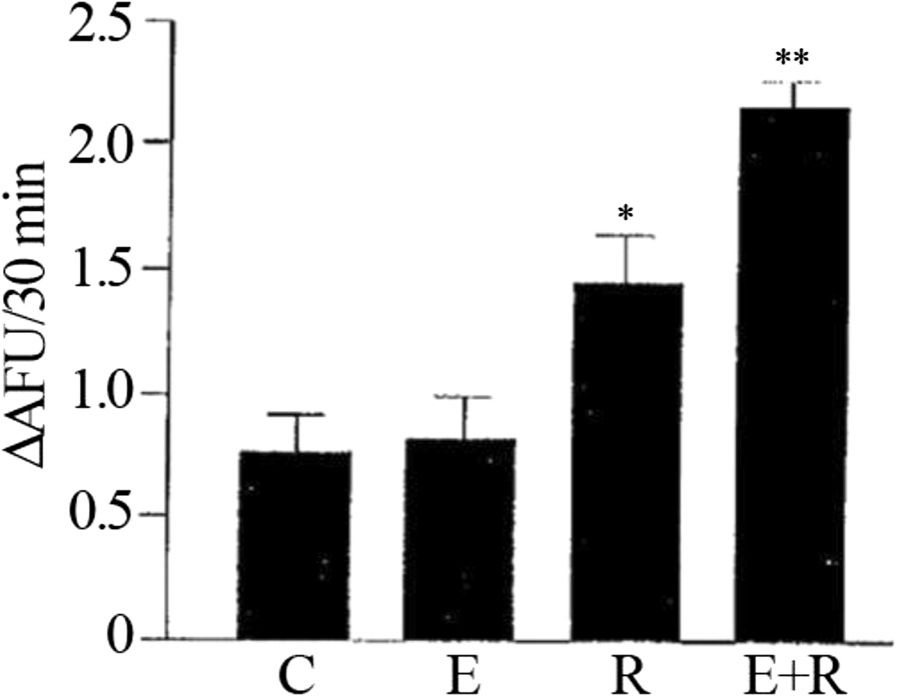
**Increase of SIRT1 enzymatic activity by combined administration of resveratrol and equol.** Fluorimetric SIRT1 activity assay to determine the effect achieved in HUVEC by the combined exposure to equol and resveratrol. Representative data of at least 3 experiments each performed in triplicate. (*= P < 0.05, **= P < 0.01). AFU: arbitrary fluorescence units; C: control; E: equol; R: resveratrol; E+R: equol + resveratrol.

It has been consistently demonstrated that activation of SIRT1 stimulates mitochondrial biogenesis and resveratrol has been utilized as a SIRT1 activator to regulate mitochondrial function [26]. Since equol exhibits a wide range of biological properties, it may be a sirtuin-targeting nutraceutical to prevent mitochondrial decline. Analysis of the effect of a combined administration of resveratrol and equol showed an increase in SIRT1 enzymatic activity in HUVEC endothelial cells (Figure 2). Although equol was much less effective to induce SIRT1, the effect of a combination of resveratrol and equol was greater than the response achieved by the single compounds (Figure 2). Therefore, the combination of resveratrol with equol is also associated with the activation of SIRT1.

### The association of resveratrol and equol activates mitochondrial biogenesis factor

**Figure 3:**
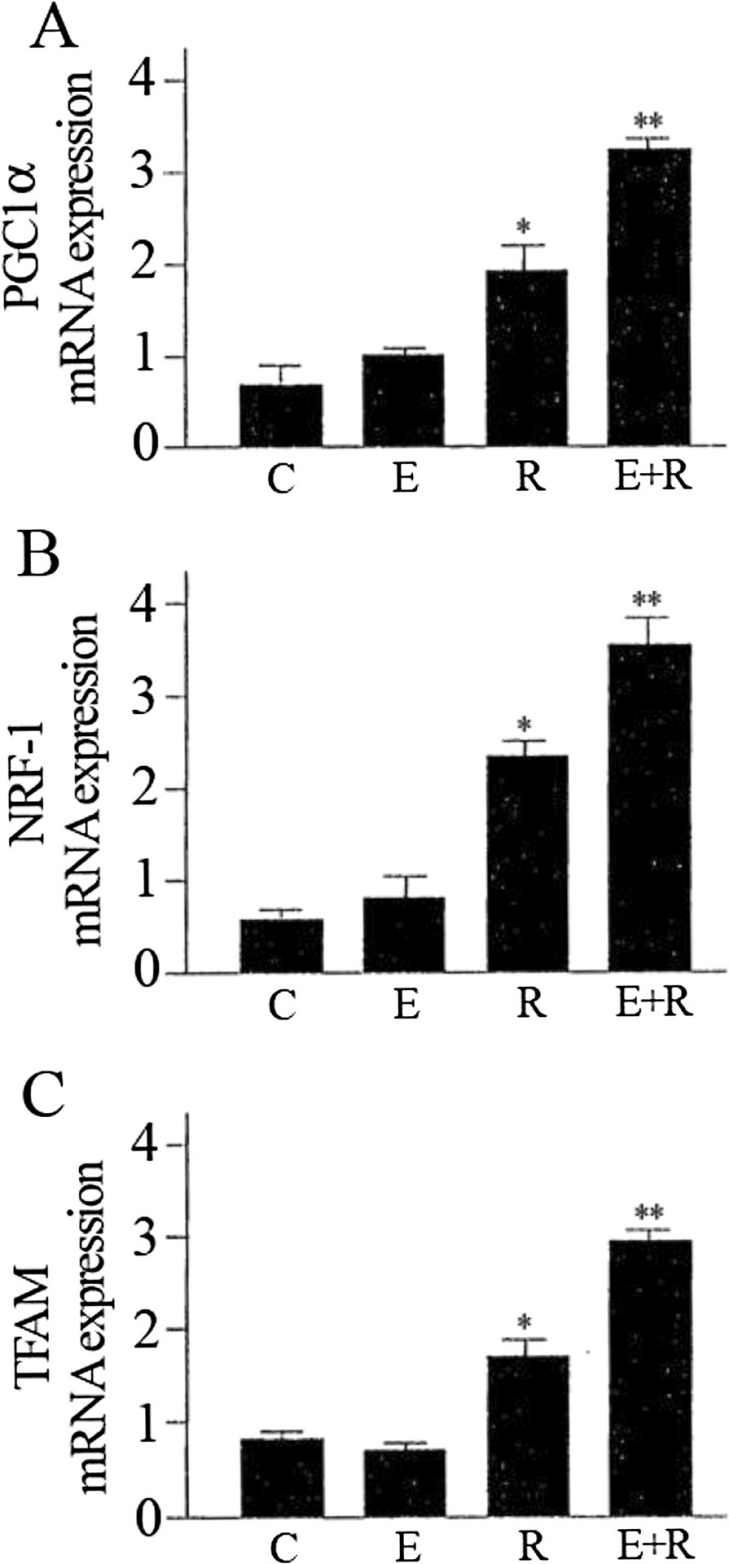
**Effect of resveratrol and equol on mRNA expression of PGC1-α (A), NRF-1 (B), TFAM (C) in HUVEC.** qRT-PCR measurement to assess the mRNA expression of the mitochondrial biogenesis factors. Representative data of at least 3 experiments each performed in triplicate. (*= P < 0.05, **= P < 0.01). C: control; E: equol; R: resveratrol; E+R: equol + resveratrol.

PGC1-α is a key component in modulating mitochondrial function and interacts with transcription factors such as NRF-1and TFAM. Specifically, PGC1-α is involved in regulating the expression of mtDNA via increased expression of TFAM which is co-activated by NRF-1 [27]. Moreover, NRF-1 in addition to regulating the activation of TFAM is also a downstream effector of SIRT1/PGC1-α [28]. Accordingly we used qRT-PCR measurements to analyse the expression of PGC1-α, NRF-1and TFAM. The results indicate that these mitochondrial biogenesis factors were strongly increased by combined treatment of resveratrol and equol (Figure 3).

## Conclusions

Collectively, these data demonstrate that the combination of two known natural products, resveratrol and equol exerts a synergistic effect on mitochondrial function because stimulates the mitochondrial biogenesis more than the single compounds alone. Clearly, more work is needed to provide novel insights into the mechanisms by which resveratrol and equol synergize to regulate the mitochondrial dynamics. However, the co-administration of these agents may be a possible nutraceutical and/or anti-ageing strategy for a number of mitochondria-associated disorders.

## Competing interests

The authors declare that they have no competing interests.

## Authors’ contributions

GS, SD and DZ conceived and designed the experiments. NS, SD performed the experiments. MV participated in the design of the experiments and critically revised the manuscript. All authors read and approved the final manuscript.
